# Emoqol-100: Development and validation of a single question for low mood in primary care. A retrospective audit.

**DOI:** 10.3399/BJGPO.2023.0011

**Published:** 2023-07-26

**Authors:** Nina Edel Dahle, Carolyn Matthew, Rachel Petronella Roskvist, Fiona Moir, Bruce Arroll

**Affiliations:** 1 Centre for Clinical Research, Uppsala University, Falun, Sweden; 2 Primary Health Care Center Britsarvet-Grycksbo, County of Dalarna, Falun, Sweden; 3 Department of General Practice and Primary Health Care, University of Auckland, Auckland, New Zealand

**Keywords:** depressive disorder, primary health care, audit, diagnostic tests, routine, general practitioners

## Abstract

**Background:**

Patients with depression need to be diagnosed and managed effectively in primary care. However, current inventories for case-finding low mood are time-consuming when considering the limited time available during appointments.

**Aim:**

To validate the diagnostic accuracy of a single question on the emotional quality of life (Emoqol-100) as a measure of depression in symptomatic patients.

**Design & setting:**

A retrospective clinical audit, validating the Emoqol-100 compared with the 9-item Patient Health Questionnaire (PHQ-9) and Burns Depression Scale Today (BDST) in South Auckland, New Zealand.

**Method:**

Consecutive patients with suspected low mood, seen over 22 months in a single primary care clinic by one of the authors, were eligible for this retrospective audit (*n* = 160). The index test was the verbally asked Emoqol-100: '*How is your emotional quality of life now, with 100 being perfect and 0 being the worst imaginable?*' The reference standard was the PHQ-9 (*n* = 426 visits) with a cut-off point of ≥10 or BDST (*n* = 513 visits) with a cut-off point of ≥6.

**Results:**

The Emoqol-100 range 0–20 had a likelihood ratio (LR) of 25.2 for low mood compared with the BDST as the reference standard; and for Emoqol-100 scores of 21–40, 41–60, 61–80, and 81–100 the LRs were 3.6, 1.7, 0.35, and 0.09, respectively. For the PHQ-9, these were 10.1, 2.9, 1.3, 0.40, and 0.2, respectively. Any score ≤60 was associated with a low mood.

**Conclusion:**

The Emoqol-100 appears to have high validity, so when it is low (≤60), it is suggestive of a high PHQ-9 or BDST score, and a mood issue probably exists. Emoqol-100 could be helpful for busy primary care professionals and other clinicians.

## How this fits in

This is the first validation study of the Emoqol-100 against the PHQ-9 for case-finding depression in primary care. It is the first derivation study of the Emoqol-100 compared with the BDST. The Emoqol-100 has a high LR when the score is low, for example ≤60, meaning the patient is likely to have a mood issue. There are significant clinical changes with the high and low LRs. For example, with the BDST the pre-test probability is 67% and for a LR of ≤20 the post-test probability of a low mood is 98%. For an LR ≥80 the post-test probability is 15%. These are clinically useful changes in the probability of low mood. It has the potential to be useful in clinical situations where low mood is suspected and time is limited, and a rapid assessment of mood would be clinically useful.

## Introduction

Depression is usually managed in the primary care setting,^1,2^ and the ability of primary care clinicians to effectively diagnose and manage depression is critically important.^3^ A meta-analysis indicated that GPs fail to diagnose more than 50% of patients with depression in their clinic, even if the diagnostic accuracy improves when the GPs meet their patients over an extended period.^4^ Those presenting with somatic symptoms, for which no apparent cause can be found, are less likely to be recognised than a similar group who present with depressive symptoms.^4^ A broad case-finding approach using a short mood inventory test can help GPs correctly identify patients with depression promptly in primary care.^5^

Depression inventories are useful in primary care to help clinicians to determine the likelihood and degree of depressive symptoms.^6,7^ The PHQ-9 has been identified as one of the most valid.^8,9^ It relates to the patient’s symptoms over the previous 2 weeks.

The Brief Mood Survey, developed by Burns, is another tool that includes three 5-item subscales for assessment of depression, anxiety, and anger during the previous 1-week period. The Brief Mood Survey has been shown to be reliable, with excellent internal consistency.^10^ There is also another shorter Burns questionnaire with five questions about the mood 'today' (on the day of administering the questionnaire), which the authors have labelled Burns Depression Scale Today (BDST) to make clear the distinction from the 1-week Brief Mood Survey. The BDST (Supplementary Table S1) can be used to assess low mood today and, to the authors' knowledge, it has not been validated apart from one conference abstract.^11^

The need for a quicker and reliable test to evaluate the severity of symptoms of low mood in primary care was identified by one of the investigators. The Emoqol-100 is a single question with a derivation validation conducted against the PHQ-9;^12^ the Emoqol-100 question is as follows: *'How is your emotional quality of life now, with 100 being perfect and 0 being the worst imaginable?*' The answer is scored verbally from 0 to 100. The Emoqol-100 question is verbally administered, takes <15 seconds to apply and interpret, and appears to be well understood by most patients.^12^

The Emoqol-100 was first used in the clinic alongside the PHQ-9. During the COVID-19 pandemic, the BDST was initiated for phone consultations, in addition to the Emoqol-100, as it was easier to administer than the PHQ-9. When time was short, only the BDST was used owing to the time needed for the PHQ-9. The PHQ-9 was also administered where possible as it was required for payment purposes, allowing comparison of the three scores.

The first study to investigate the value of a test is called the derivation study, while the second validation study should validate the derivation findings in a different population. The studies should be performed according to the STARD (Standards for Reporting of Diagnostic Accuracy Studies) statement,^13^ and the final test of a diagnostic test or model should investigate whether it is accurate and generalisable enough for the purpose for which it was derived.^14^ Criterion validity involves comparison with a gold standard, which is called concurrent validity when the comparison is made simultaneously.^15^ These validation and/or derivation studies are ideally done in the settings where the diagnostic test will be used.^16^

This article aimed to validate the findings in the derivation study of Emoqol-100 and PHQ-9 by validating the Emoqol-100 against PHQ-9 as a reference standard, and to derivate the Emoqol-100 against the BDST questionnaire.

## Method

A retrospective audit was conducted over 22 months, from 25 November 2002—28 September 2022, at a general practice clinic in South Auckland, New Zealand. Participants were consecutive patients (*n* = 160) seen by one of the authors, in whom low mood was a key issue and who were coming for fully funded wellness visits. A patient requests a wellness visit for a 30-minute consultation, usually for emotional distress. The clinic gets paid an increased fee for the service by the health system. Some of these patients were regular, and clinic colleagues referred others for a Focused Acceptance and Commitment Therapy (FACT) consultation.^17^ Patients were eligible for the audit if they had a recorded Emoqol-100 score and a PHQ-9 score or BDST questionnaire administered at the same visit. These were assessed during the visit by the GP. The Emoqol-100 was the index test, and the BDST questionnaire or PHQ-9 was the reference standard. The order of doing the Emoqol-100, the BDST questionnaire, and the PHQ-9 were variable, but the Emoqol-100 was generally done first as it was the quickest to complete and on some occasions, the only one done. The reference tests (PHQ-9 and BDST) were not given blindly to the patients. While the clinician had other information, such as medication and medical history, this did not alter the administration of the Emoqol-100 test, BDST, or the PHQ-9. Only patients with reasonably good English language abilities were asked the Emoqol-100.

The analysis was done according to the method of Guyatt and Rennie for calculating LRs. For each level of the Emoqol-100, the true positive number is divided by the total depressed and the false positive divided by the total not depressed (TP/all depressed) / (FP/those not depressed).^16^ A likelihood ratio >1 increases the post-test probability of the condition, while a likelihood ratio <1 decreases the post-test probability of the condition.

A recent meta-analysis reported that a PHQ-9 score of ≥10 is the level where the combination of sensitivity and specificity is maximised overall, and this was the cut-off used.^9^ For BDST, a score ≥6 is classified as depression and was used as a cut-off.^18^ The number of patients available determined the sample size during the study period. There was no public or patient involvement in this work.

## Results

### Baseline characteristics

**Figure 1.**
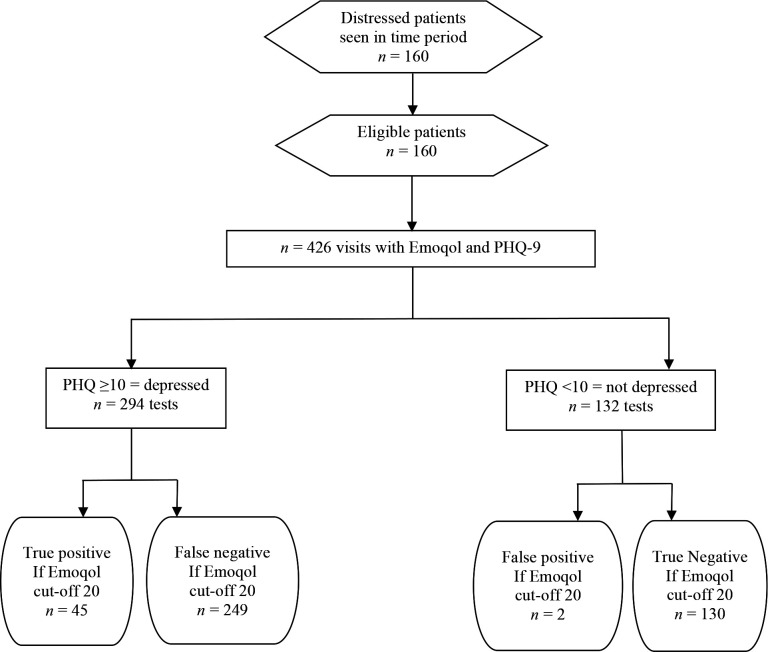
Flow diagram for records of reference standard PHQ-9 and Emoqol-100 score (*n* = 426 visits)

There were 160 patients and *n* = 426 visit records of PHQ-9 and *n* = 513 visit records of BDST. The findings are shown in Figures 1 2. The majority of the patients (62%) were women, and the median age was 35 years (14–78), as shown in Table 1. The distribution of ethnic groups reflected the general population of the clinical study site reasonably well. The median score of Emoqol-100 was 55. The median for PHQ-9 was 13, which is equal to mild-to-moderate depression, and the median for BDST was 8, equivalent to moderate-to-low mood. Low mood was present in 69% of the sample according to PHQ-9, and in 67% according to BDST. The practice had 5000 registered patients. There were seven GPs, one GP trainee, two nurse practitioners who work as GPs four nurses, and two healthcare assistants. The practice is called a very low-cost access (VLCA) practice, meaning that more than half the patients are either from Maori or Pacific ethnic groups, or live in the most socioeconomically deprived quintile. Because it is a VLCA practice, the clinic gets more funding from the health system.

**Figure 2.**
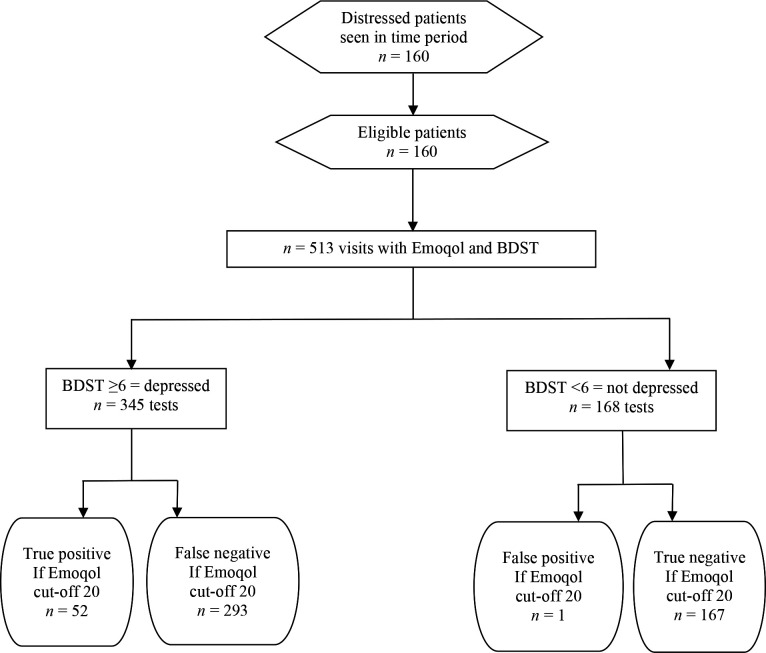
Flow diagram for records of reference standard Burns Depression Scale Today (BDST) and Emoqol-100 score (*n* = 513 visits)

**Table 1.** Baseline measures (*n* = 160 patients), Burns Depression Scale Today (*n* = 523), PHQ-9 questionnaires (*n* = 434)

Age, median (SD)	35 years (±15.9)
Gender	Female: 100 (62%)Male: 60 (38%)
Ethnic group	European: 79 (49%)Māori: 38 (24%)Indian: 13 (8%)Samoan: 8 (5%)Other: 22 (14%)^a^
BDST	Range (20–0)Median: 8
PHQ-9	Range: (27–0)Median: 13
Emoqol score	Range: (0–100)Median: 55

^a^Asian, Chinese, Cook Island Māori, Latin American, Middle East, Fijian, Tongan, Niuean.

BDST = Burns Depression Scale Today. PHQ-9 = Patient Health Questionnaire 9-item.

### Emoqol-100 validation against PHQ-9

For patients with an Emoqol-100 score of ≤20, the LR was 10.1, with a positive PHQ-9 (≥10) as the reference standard (Table 2). The Emoqol-100 score 21–40 had an LR of 2.9, score 41–60 had an LR of 1.3, score 61–80 had an LR of 0.40, and a score of 81–100 had an LR of 0.2 (Table 2). Based on the PHQ-9 ≥10, the highest positive predictive value was 96% for a cut-off point of ≤20 (Table 2). This means that a patient who scores ≤20 is 96% likely to have a PHQ-9 score of ≥10, indicating a high probability of a low mood at that visit and a clinically significant increase from the average low mood of 69%.

**Table 2.** Validation assessment of Emoqol-100 with reference standard PHQ-9

Emoqol-100		
Range	Likelihood ratio positive	Positive predictive value^a^
0–20	10.1	96%
21–40	2.9	87%
41–60	1.3	74.5%
61–80	0.4	47.4%
81–100	0.2	15%

^a^Also known as the post-test likelihood of a positive test. Likelihood ratio positive = sensitivity/(1-specificity) (these scores are >1.0). Likelihood ratio negative = 1-sensitivity/(specificity) (these scores are <1.0). PHQ-9 = 9-item Patient Health Questionnaire.

### Emoqol-100 derivation against Burns Depression Scale Today

For patients with an Emoqol-100 score of ≤20, the LR of low mood is 25.2, with a positive BDST (≥6) as the reference standard (Table 3). The Emoqol-100 score 21–40 had an LR of 3.6, score 41–60 had an LR of 1.7, score 61–80 had an LR of 0.35, and score 81–100 had an LR of 0.09 (Table 3). As the Emoqol-100 score gets higher, the LR drops, and in the higher Emoqol-100 range, the patient is much less likely to suffer from a mood disorder. Based on the BDST ≥6, the highest positive predictive value was 98% for a cut-off point of ≤20 (Table 3). This means that a patient who scores ≤20 is 98% likely to have a BDST score of ≥6, which is a clinically significant increase from the average low mood on the BDST of 67%. The same applies for a Emoqol-100 score of >80; the post-test likelihood of this is a positive predictive values of 15%, which is a clinically significant decrease from 67%.

**Table 3.** Derivation assessment of Emoqol-100 with reference standard Burns Depression Scale Today (BDST)

Emoqol-100		
Range	Likelihood ratio positive	Positive predictive value
0–20	25.2	98%
21–40	3.6	88%
41–60	1.7	78%
61–80	0.35	41%
81–100	0.09	15%

### Emoqol-100 against PHQ-9 Burns Depression Scale Today as a receiver operating curve

**Figure 3.**
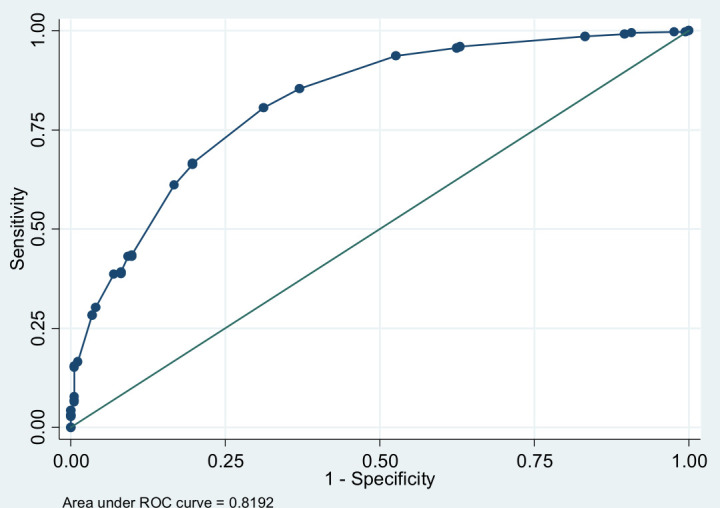
Emoqol (continuous) as predictor of Burns Depression Scale Today (BDST) ≥6. (area under curve 0.8192 [95% confidence interval = 0.78078 to 0.85762]).

The Emoqol-100 (continuous) as a predictor of the PHQ-9 ≥10 had an area under the curve (AUC) 0.7698 (95% confidence interval [CI] = 0.72270 to 0.81698; not shown). An AUC of 0.7–0.8 is considered to be good. For the Emoqol-100 (continuous) as a predictor of the BDST ≥6, the AUC was 0.8192 (95% CI = 0.78078 to 0.85762; Figure 3). An AUC 0.8–0.9 is considered excellent. The receiver operating characteristic [ROC] curves were done with Stata (version 17).

## Discussion

### Summary

The Emoqol-100 was validated against the PHQ-9 and the results were consistent with the previous derivation study.^12^ The Emoqol-100 score in the low range is associated with a high PHQ-9 and a high BDST (high scores indicate low mood). The higher the Emoqol-100 score, the lower the scores for PHQ-9 and BDST. The Emoqol-100 score in the low range is associated with an increased risk of low mood, according to both the PHQ-9 and the BDST. For an Emoqol-100 score of 0–20, the LR for PHQ-9 is 10.1 and for BDST is 25.2, which is very high and suggests that the test will significantly change post-test probabilities from pre-tests. It is unusual to have such high LRs in clinical medicine.^19^ In addition, the Emoqol-100 with scores 81–100 have very low LRs, and a high score (a negative result) could be used to rule out low mood.

### Strengths and limitations

This is a clinical audit and, as such, is a pragmatic study applied to consecutive patients with known or suspected depression. The advantage of an audit is that it is possible to measure inventories while in use in regular clinical practice, without the confounding issues of consent and information sheets, which can cause a selection bias in planned research. The Emoqol-100 has been validated and derivated in the clinical setting where it is intended to be used. The prevalence of low mood was significantly higher than in a consecutive series of patients seen in a usual general practice setting. This sample of included patients was seen by one of the authors for extra mental health care. This should not affect the properties of the test, but rather the interpretation of a negative test since a diagnosis is hard to rule out in a highly prevalent condition. This study has several limitations. The clinical audit only applied to one practitioner, and it was impossible to blind the measurement of the reference standard PHQ-9 or BDST. The PHQ-9 is becoming the common tool for primary care assessments of depression.^20^ The Emoqol-100 measures the mood right now, and that can be considered both a weakness and a strength. It may be less valid than tools measuring more extended periods of low mood, but may potentially be more precise and faster to complete, as well as not being subject to recall bias.

### Comparison with existing literature

Low mood was present in the cohort for 69% for PHQ-9 and 67% for BDST. This is a very high prevalence, and thereby pre-test probability is high compared with the average primary care prevalence of mood disorders of approximately 12.9%.^21^ The prevalence was expected to be high as the patients were selected for extra attention for their mental health. The prevalence should not affect the LR results since it is a function of the sensitivity and specificity.^22^ Diagnostic properties like sensitivity and specificity are not influenced by changes in disease prevalence itself, whereas positive and negative predictive values directly depend on prevalence.^23^ The Emoqol-100 is not intended as a screening tool but needs to be evaluated in a screening setting.

The PHQ-9 takes around 5 minutes to complete and has been identified as a useful screening tool for depression.^4,24^ However, using tools that take several minutes to apply and interpret may be impractical in a standard primary care setting, where consultations often are very short. Emoqol-100 is feasible, fast, easy, and helpful both in monitoring severity and for following patients over time. Table 4 shows the range of Emoqol-100 scores, and the median values of those ranges on the PHQ-9 and the BDST. The authors hope this is helpful for clinicians when seeing what equivalent scores would be for the Emoqol-100.

**Table 4.** Emoqol-100 versus PHQ-9 and BDST

Emoqol 0–100 range	PHQ-9 median range 0–27	BDST median range 0–20
0–20	17	15
21–40	16	11
41–60	15	6
61–80	9	4
81–100	6	1

### Implications for research and practice

The Emoqol-100 is the briefest of all mental health tools. It takes <15 seconds to verbally administer and interpret and has the advantage of patients not having to read. The authors speculate that by verbally asking the Emoqol-100 rather than doing a written questionnaire, the opportunity for the patient to be seen, heard, and understood is increased, thereby enhancing beneficial patient-centred communication.^25,26^ The Emoqol-100 may also be helpful for monitoring mood over time: since it specifically assesses the mood right now, there should be no recall bias.

There are significant clinical changes with the high and low LRs. For example, with the BDST the pre-test probability is 67% and for a LR of ≤20, the post-test probability of a low mood is 98%. For a LR >80, the post-test probability is 15%. These are clinically useful changes in condition probability. It has the potential to be useful in clinical situations where low mood is suspected and time is limited, and a rapid assessment of mood would be clinically useful.

The authors suggest that the Emoqol-100 will be a valuable tool for clinicians in other clinical situations when there is not enough time to do a longer inventory, such as when emotional issues are raised near the end of a consultation or during a ward round. A score of ≤60 would indicate the need for further help and a longer appropriate intervention made at that visit or a subsequent appointment. For those scoring >60, the two Whooley questions on the PHQ-2 about feeling depressed or losing pleasure during the last month could be asked; negative answers would most certainly rule out depression.^27^

Future research could systematically interview primary care clinicians and patients to ask how they feel about being asked these questions and what they think are the strengths and limitations. The authors' anecdotal experience of teaching the Emoqol-100 to groups of primary care clinicians is that they are highly positive about it. They realise that if the Emoqol-100 is inconsistent with the clinical picture or history, they can do a PHQ-9 or a BDST longer questionnaire.

For clinical practice, the US Preventive Services Task Force (2016) recommends the following: *'Commonly used depression screening instruments include the Patient Health Questionnaire (PHQ) in various forms and the Hospital Anxiety and Depression Scales in adults, the Geriatric Depression Scale in older adults, and the Edinburgh Postnatal Depression Scale (EPDS) in postpartum and pregnant women.* ^28^ *All positive screening results should lead to an additional assessment that considers the severity of depression and comorbid psychological problems (eg, anxiety, panic attacks, or substance abuse), alternate diagnoses, and medical conditions.*'

In conclusion, the Emoqol-100 is a case-finding tool that has now been validated against the PHQ-9 and derived against the BDST. It could be used when the pre-test probability of a low mood is higher than usual, as in patients complaining of possible mood-related symptoms such as sleep difficulties, pain, and fatigue.^29^ The LRs at very high and very low Emoqol-100 scores are robust, creating clinically significant changes in post-test probabilities, which is what is required in diagnostic tests.

The Emoqol-100 is the briefest of all mental health tools known to the authors. It and the BDST are the only inventories to measure how the patient is feeling today. The Emoqol-100 has the advantage of patients not having to read or interpret a written or verbally asked questionnaire. It takes only seconds to administer, which could be highly useful in case-finding in primary care. Further research is required using the Emoqol-100 in different clinical settings, such as screening in primary care and clinical use in secondary care by other clinicians, and as a tool for monitoring the ongoing mood state.

## Data

Anonymous data is available on reasonable request. A copy of the BDST can be seen in Supplementary Table S1.
